# Obstructive sleep apnea and neurodegenerative diseases: A bidirectional relation

**DOI:** 10.1590/S1980-57642015DN91000003

**Published:** 2015

**Authors:** Christianne Martins Corrêa da Silva Bahia, João Santos Pereira

**Affiliations:** 1 Setor de Distúrbios do Movimento/ Neurologia/ Hospital Universitário Pedro Ernesto/ Universidade do Estado do Rio de Janeiro(UERJ).; 2 Pós Graduação Stricto Sensu em Ciências Médicas/Faculdade de Ciências Médicas/ Universidade do Estado do Rio de Janeiro.; 3 Serviço Interdisciplinar de Medicina do Sono/Hospital Universitário Pedro Ernesto/ Universidade do Estado do Rio de Janeiro

**Keywords:** obstructive sleep apnea, sleep disorder, neurodegenerative diseases

## Abstract

Sleep disorders are common during the clinical course of the main neurodegenerative diseases. Among these disorders, obstructive sleep apnea has been extensively studied in the last decade and recent knowledge regarding its relationship with the neurodegenerative process points a bidirectional relationship. Neurodegenerative diseases can lead to functional changes in the respiratory system that facilitate the emergence of apnea. On the other hand, obstructive sleep apnea itself can lead to acceleration of neuronal death due to intermittent hypoxia. Considering that obstructive sleep apnea is a potentially treatable condition, its early identification and intervention could have a positive impact on the management of patients with neurodegenerative diseases.

## Abstract (pt)

Os distúrbios do sono são comuns ao longo do curso clínico das principais doenças neurodegenerativas. Dentre estes, a apneia obstrutiva do sono tem sido muito estudada na última década e avanços no conhecimento sobre sua relação com o processo neurodegenerativo tem apontando para uma relação bidirecional. As doenças neurodegenerativas podem levar a alterações funcionais no sistema respiratório que facilitam o surgimento da apneia, assim como, a própria apneia obstrutiva do sono, ao causar hipóxia intermitente, parece acarretar na aceleração do processo de morte neuronal. Considerando que a apneia obstrutiva do sono é uma condição potencialmente tratável, sua identificação e intervenção precoces podem ter impacto positivo no manejo de pacientes com doenças neurodegenerativas.

## INTRODUCTION

Neurodegenerative diseases are characterized by progressive and inexorable neuronal loss, manifesting clinically through gradual impairment of cognitive, psychic and/or motor domains with different degrees of severity and age of onset depending on the specific condition presented. The cause of neurodegeneration varies with disease, but the inability of cells to fold specific proteins in their original conformation, resulting in abnormal accumulation in the form of fibrillar aggregates or inclusion bodies, seems to be a physiopathological mechanism common to the majority of these conditions^1^ (Table 1).

**Table 1:** General characteristics of main neurodegenerative diseases.

Disease	Principal mechanism	Preferential location	Authors
Alzheimer’s disease	Senile plaques: Aβ protein deposits Neurofibrillary tangles: Intracellular accumulation of p-tau	Hippocampus	Jellinger KA (2012)^1^
Parkinson’s disease	α-synuclein accumulation, Lewy body cytoplasmic inclusions	Brain stem, mainly Striatonigral dopaminergic system	Jellinger KA (2012)^1^
Lewy body disease	α-synuclein accumulation, Lewy body cytoplasmic inclusions	Wide distribution, mainly frontal cortex, brain stem, basal prosencephalon, cortical areas with limbic projections, dorsal efferent nucleus of the vagus	Beyer K et al. (2009)^2^
Multiple system atrophy	α-synuclein accumulation, glial cytoplasmic inclusions	Striatonigral and olivopontocerebellar system	Ahmed Z et al. (2012)^3^
Huntington’s disease	Huntingtin intranuclear inclusions	Caudate nuclei and putamen	Ahmed Z et al. (2012)^3^
Frontotemporal dementia	Accumulation of the tau protein, ubiquitin and TDP-43 immunoreactive inclusions	Frontal and temporal lobes	Seelaar H et al. (2011)^4^
Amyotrophic lateral sclerosis	Immunoreactive ubiquitin and TDP-43 inclusions Bunina body cytoplasmic inclusions	Motor neuron	Wijesekera LC et al. (2009)^5^

Aβ: Amyloid βeta protein; p-tau: tau hyperphosphorylated protein; TDP-43:TAR DNA-binding protein of 43 kDa.

Although under-emphasized, sleep is impaired in the main neurodegenerative diseases.^6,7^ In Alzheimer's disease (AD), approximately a quarter of patients have disrupted circadian rhythm leading to sleep fragmentation, increased daytime napping and the "sundowning" phenomenon, characterized by a confusional state which occurs at night fall.^8,9^ In Parkinson's disease (PD), 40-90% of patients have sleep-related problems.^10,11^ These disturbances include REM sleep behavior disorder (RBD), found in 15-33% of patients.^10,12^ RBD is currently considered a predictor of the development of PD and other synucleinopathies. Prospective studies have shown that approximately 80% of patients with idiopathic RBD go on to present one of these conditions within two decades.^14,15^

The relationship between obstructive sleep apnea (OSA) and neurodegenerative diseases remains less clear, but has been the focus of extensive study. The latest literature on the theme points to a bidirectional relationship, with one condition interfering with the other and vice-versa.

**Obstructive sleep apnea - Preliminary considerations.** OSA is characterized by repetitive episodes of obstruction of the upper airway during sleep, causing a total or partial limitation of air flow, despite continued effort from the respiratory muscles. The main consequences are: intermittent hypoxia, sleep fragmentation, hypercapnia and sympathetic hyperactivity. OSA is considered an independent risk factor for developing arterial hypertension^16^ and also appears to be implicated in increased risk of cardiovascular diseases,^16^ stroke,^16^ insulin resistence,^16^ cognitive deficit,^17^ white matter change,^18^ anxiety^19^ and depression.^19,20^

The prevalence of OSA is estimated at 17-26% in men and 9-28% in women.^21^ In Brazil, a recent study involving 1042 participants from São Paulo city aged 20-80 years, showed that 32.8% presented criteria for OSA.^22^ The chances of developing OSA increases with age, being greater in individuals over 60 years of age.^21,22^ Obesity and enlarged neck circunference are also risk factors for OSA.^23^

The physiopathology of OSA is believed to involve mechanisms which increase the collapsibility of the pharynx, due to anatomical changes or dysfunction in neuromuscular control of the upper airway.^16,24^

The gold standard for diagnosing OSA is use of polysomnography performed in a sleep laboratory with neurological and respiratory monitoring throughout the night. The diagnostic criteria for OSA were recently revised^25^ in order to embrace the latest discoveries about the disease. The current diagnostic criteria and classification of OSA in adults are given in Table 2.

**Table 2:** Diagnostic criteria and classification of OSA in adults.

Diagnostic criteria (ICSD-3) (A+ B) or C		Classification (AASM Task Force)^26^
**A) Clinical. Presence of one or more of the following** 1) Complaint of sleepiness, non-restorative sleep, fatigue or insomnia 2) Complaint of awakenings with sensation of breath holding, gasping or choking 3) Reports by observers of snoring or breathing interruptions 4) Diagnosis of hypertension, mood disorder, cognitive deficit, coronary artery disease, stroke, congestive heart failure, atrial fibrillation or diabetes mellitus type 2	**B) Polysomnographic** 1) Five or more predominantly obstructive respiratory events (obstructive or mixed apneas, hypopneas or RERA), per hour of sleep	A) Mild: ≥5 and <15 events/hour of sleep B) Moderate: ≥15 and <30 events/hour of sleep C) Severe: ≥30 events/hour of sleep
	**C) Polysomnographic** 1) Fifteen or more predominantly obstructive respiratory events (obstructive or mixed apneas, hypopneas or RERA), per hour of sleep	

ICSD-3- International Classification of Sleep Disorders-Third Edition; AASM: American Academy of Sleep Medicine; RERA: respiratory effort related arousals.

In general, the treatment of choice for OSA is the use of CPAP (continuous positive airway pressure). Other treatment modalities include intra-oral devices, mandibular advancement surgery and otorhinolaryngologic surgery, the indication for which must be assessed within the clinical context of each patient.^26^

**Central respiratory control and changes with aging.** Specific groups of neurons in the brain stem have rhythmic firing activity during respiration. For simplicity's sake, these can be divided into two groups: those more important during the inspiratory phase (pre-Bötzinger complex and rostro-ventral respiratory group) and those more active during the expiratory phase (Bötzinger complex). These groups of neurons form synapsis among each other and with cranial and spinal motoneurons which, in turn, convey efferences to the respiratory muscles (e.g. diaphragm and intercostal muscles) and muscles regulating upper airway patency (e.g. genioglossus muscle).^27,28^

The rhythmic activity of these neurons provides the basal respiratory pattern, effective for adequate gaseous exchange between the lung and atmospheric air under normal resting conditions. A complex network of connections among these neurons and with others from the cortex, cardiovascular, visceral, autonomic and skeletal muscle systems allow changes in respiratory activity according to the situation, thus maintaining arterial and tissue pH, CO_2_ and O_2_ within normal levels. Hence, postural changes, phonation, swallowing, physical activity and the transition between sleep and wake states, triggers changes in respiratory drive.^29^

These changes are carried out via two pathways: the ascending reticular activating system, which is more active during the awake state; and by chemoreceptors sensitive to changes in pH, CO_2_ and O_2_, which act by promoting involuntary changes in respiration.^29^ Thus, falls in pH and O_2_ levels and increases in CO_2_ in arterial blood, lead to a chain of signalling which increases the excitatory activation in motoneurons which control the breathing muscles, promoting hyperventilation. When normality is reestablished, the excitatory stimulus of these motoneurons ceases with consequent reduction in respiratory drive and return to basal activity.

The main neurotransmitters involved in central respiratory control are glutamate,^30^ gamma-aminobutyric acid (GABA) and glycine.^31^ More recent studies however, have shown that acetylcholine and serotonin can play a key role in the modulation of muscle tonus of the upper airway^26^ and in activity of the laryngeal dilatator muscles, respectively.^32,33^

With aging, there is a lower ventilatory response to hypoxia and to hypercapnia.^34^ This phenomenon appears to be related both to reduced sensitivity of chemoreceptors and to structural changes in the respiratory apparatus with age, leading to decreased motor performance of the respiratory muscles in response to stimuli from motoneurons.^29^ Advanced age also promotes anatomical and functional changes in the upper airway which increase predisposition to collapse. These changes include greater surrounding soft tissue, narrowing the lumen, and reduced negative pressure reflex, a protective reflex of the upper airway which activates its dilatator muscles in the presence of negative pressure so as to prevent airway closure.^24,29^ Consequently, a group of conditions predisposes elderly to developing OSA.

**Neurodegenerative diseases and OSA.** Neurodegenerative diseases typically affect the more elderly,^35,36^ i.e. the population group most susceptible to obstruction of the upper airway. However, the high rate of OSA in AD (53.9%),^37^ PD (27-60%)^38,39^ and multiple system atrophy (MSA) (37%),^40^ suggests that specific mechanisms of the neurodegenerative process combine with normal aging-related changes in the respiratory system to promote the development of OSA in this patient group.

The objective of the present article was to review the latest literature focusing on novel proposed explanations of the relationship between the two conditions.

## METHODS

A search of the digital databases Pubmed, Scielo and Lilacs was performed using the descriptors "apneia obstrutiva do sono", "distúrbios do sono" and "doenças neurodegenerativas", along with their equivalent terms in English: "obstructive sleep apnea", "sleep disorders" and "neurodegenerative diseases", encompassing all publications spanning the period from January 2004 to September 2014 relevant to the study purpose.

Review articles, systematic reviews and original articles addressing the relationship between OSA and the main neurodegenerative diseases of the central nervous system (CNS), including basic knowledge of physiology as well as clinical presentation and repercussions of treatment of one disease on another, were retrieved. Thus, studies were selected on AD, PD and MSA, with the latter included given the importance of OSA in the clinical evolution of MSA.

Emphasis was given to more recent studies (last 5 years). However, highly relevant older articles, such as population-based studies involving a large number of participants, were also featured. Studies not pertinent to the proposed theme, uncontrolled clinical trials and case reports, were excluded.

## RESULTS

Of the 118 articles retrieved, 15 were excluded for not being in Portuguese or English, and 53 because they were not directly related to the proposed theme or failed to address the neurodegenerative diseases of the CNS cited. A further 7 studies were excluded for being case reports or uncontrolled clinical trials.

**Alzheimer's disease and OSA.** AD is the most prevalent neurodegenerative disease worldwide. In 95% of cases, the disease occurs in its sporadic form, where environmental factors are believed to play a key role in triggering the neurodegenerative process.^41^

Intermittent hypoxia, a consequence of OSA, has been implicated as the one of the main environmental factors involved in the emergence of AD, by promoting the expression of genes related to inflammation and cellular aptosis.^40^ Intermittent hypoxia promotes the activation of BACE1 (β-site amyloid precursor protein cleaving enzyme),^41^ responsible for cleavage of the amyloid precursor protein (APP) in β amyloid species (Aβ) accelerating the accumulation of the substance in the CNS. In addition, hypoxia is involved in increased hyperphosphorylation of tau protein,^41,42^ impairment of the blood-brain barrier, activation of pro-inflammatory pathways with consequent production of reactive oxygen species and, according to the latest evidence, in neuronal apoptosis.^43,44^ Animal models, when submitted to repeated hypoxia, exhibited neuronal apoptosis in the CA1 region of the hippocampus, a key area involved in memory consolidation.^45^ Other brain regions such as the frontotemporal cortex, locus ceruleus, limbic system, cerebellum and brain stem, also appear to be affected by intermittent hypoxia.^41^

Another point of convergence between AD and OSA is the genetic predisposition that both share through the APOEε4 gene. Carriers of the APOEε4 gen are at greater risk of developing both AD and OSA,^46^ while moderate-severe OSA patients with APOEε4+ have worse performance on memory and executive function tests compared to OSA patients carrying APOEε4-.^47^

Finally, cholinergic transmission deficit found in AD can predispose patients to developing apnea, since cholinergic activity influences the modulation of muscle tonus of the upper airway. Central inhibitors of acetylcholinesterase such as donepezil were shown to reduce the apnea and hypopnea index (AHI) in patients with AD and OSA.^48^ However, there is insufficient evidence to indicate the use of this medication for the treatment of OSA in these patients.^49^

**Parkinson's disease and OSA.** PD is the second-most-common neurodegenerative disease.^36^ Data from the literature attempting to establish a relationship between PD and OSA are conflicting. Some authors have found an association between the degree of motor deficit in PD and the severity of OSA,^38,39^ while others have failed to confirm this association.^50,51^

However, the knowledge that PD patients have major functional changes in the respiratory system is well-documented in the literature. Studies employing spirometry have shown a high prevalence of upper airway obstruction in patients with PD (24-65%),^52-54^ which can be alleviated by Levodopa.^55^ Direct visualization of the upper airway of patients with Parkinsonism using fiber-optic endoscopy has revealed involuntary contractions of the glottic and subglottic structures.^54^ Parkinsonians also progress with significant restrictive ventilatory disturbance (28-70%),^53,56^ which leads to reduced lung volume during inspiration and resultant reduction in caudal traction of the trachea and in dilatation of the pharynx.^57^ These data suggest dysfunction in the upper airway muscles and ribcage of PD patients, possibly secondary to the tremor, rigidity and bradykinesia associated with the disease. Whether these alterations are related to an increased risk of OSA remains unclear.

Liancai et al.^58^ described the accumulation of alpha-synuclein in the vagus nerve and its pharyngeal branch in patients with PD. The vagus nerve and particularly its pharyngeal branch are important in motor innervation of the muscles of the larynx, pharynx and some palate muscles. The study found a correlation between density of alpha synuclein in nerve fibers and degree of dysphagia. In the literature searched no data about the correlation of this finding with the presence and severity of OSA was found.

During the course of PD, degeneration of serotoninergic neurons also occurs,^59^ important for maintaining the patency of the upper airway, where its absence can contribute to pharyngeal collapse.^33^ This mechanism however, does not appear to play a significant role in the development of OSA in PD patients.^60^

Patients with OSA and PD have a different clinical profile to OSA patients without PD. Parkinsonians generally have a lower body mass index^61,62^ and less marked falls in saturation of oxyhemoglobin during apnea and hypopnea events.^39,62,63^ Excessive daytime sleepiness, an important symptom of OSA, was not correlated with AHI in PD patients.^50,51^ Recent metanalyses suggest that parkinsonians do not have a greater risk of developing OSA compared to controls,^61,63^ but acknowledged the limitation of studies in reaching definitive conclusion on the relationship between PD and OSA.

**Multiple system atrophy and OSA.** Multiple System Atrophy (MSA) is characterized by a combination of parkinsonianism, cerebellar, dysautonomic and pyramidal features, in which respiratory disturbances such as OSA, stridor and central apnea represent important features of clinical evolution.^65^

Visualization of the upper airway in MSA patients using fibre-optic laryngoscopy has shown narrowing of the airway at the level of the vocal folds, base of the tongue and soft palate,^66^ as well as rhythmic, bilateral contractions of the arytenoids^54,66^ plus the presence of "floppy epiglottis" ^66^ - a condition in which the epiglottis is sucked into the glottis during inspiration. As in PD, these findings suggest dysfunction of the muscles of the upper airway due to parkinsonism symptoms of the disease.

Besides these changes, degeneration of the serotoninergic and cholinergic system are found in MSA,^68^ both important neurotransmitters involved in the respiratory physiology, whose deficiency can lead to the risk of developing OSA in these patients.^33,68^

**Use of CPAP in neurodegenerative diseases.** Numerous studies have compared the cognitive performance of OSA patients, with and without the use of CPAP, many of which reported improved cognitive function in the group in use of CPAP.^17,69-71^ However, only one study involving a small number of patients has objectively shown morphological changes in brain gray matter in OSA patients after intervention with CPAP.^72^

In patients with OSA and AD, adherence to CPAP appears to slow cognitive decline, particularly in the executive function domain, and also stabilize depression symptoms and enhance sleep quality.^73,74^

Among patients with OSA and PD, the use of CPAP reduced the number of nighttime awakenings and episodes of excessive daytime sleepiness, and also increased the percentage of deep sleep stages, translating to improved sleep quality in these patients.^75^

In MSA associated with OSA, CPAP also proved effective in reducing AHI, although should be used with caution in patients with "floppy epiglottis" given the increased risk of exacerbating the obstruction. CPAP is also considered the treatment of choice for stridor, another common respiratory disorder associated with mortality risk in MSA patients.^65^

## Conclusion.

The relationship between OSA and the neurodegenerative process is not fully elucidated. However, evidence found in the literature to date points to a bidirectional relationship, akin to a two-way path.

The neurodegenerative process can facilitate the emergence of OSA by triggering functional alterations in the respiratory apparatus which promote obstruction of the upper airway, such as those outlined in PD and MSA. Similarly, some neurodegenerative diseases exhibit acetylcholine and serotonin deficit, important neurotransmitters involved in maintaining patency of the upper airway. OSA on the other hand, through intermittent hypoxia, facilitates neurodegeneration by promoting expression of the genes linked to inflammation and neuronal apoptosis.

Since no curative treatment for neurodegenerative diseases is currently available, the early detection and intervention of OSA can have a positive impact on the clinical management of these patients. The challenge for the coming decade is to continue the advancement of knowledge on this relationship, promoting studies which better assess the mechanisms involved, the importance of interaction with genetic and environmental factors, determinants for reversibility of neuronal damage, and the impact of treating one condition on the evolution of another.
